# More than Just Aversive: A Network Analysis of the Dark Triad, Coping, and Psychopathology

**DOI:** 10.3390/bs15121617

**Published:** 2025-11-24

**Authors:** Micheala McIlvenna, Tayler Truhan, Kostas Papageorgiou

**Affiliations:** School of Psychology, Queen’s University of Belfast, Belfast BT9 5BN, UK

**Keywords:** dark triad, network analysis, mental health

## Abstract

The dark triads are a set of personality traits (subclinical narcissism, subclinical psychopathy, and Machiavellianism) aggregated due to their primarily socially aversive associations. However, recent work has suggested that some dimensions of these traits (e.g., narcissistic extraversion) may be adaptive in coping with psychopathology. Lesser researched are the dimensions of psychopathy and Machiavellianism in association with mental health and coping. The current study, therefore, examined the dimensions of all dark triad traits in association with psychopathology. Participants (*N* = 350) completed questions on dark triad factors, psychopathology, and coping. Data were analyzed using lasso regularized network analysis. The findings demonstrated that narcissistic extraversion and neuroticism could act positively and negatively, respectively, on depression through Machiavellian agency. Machiavellian agency also connected strongly and negatively to depression; however, centrality indices showed that this connection was not influential. Machiavellian agency instead acted as a bridge node to facilitate the indirect, negative connection from narcissistic extraversion and the positive connection from narcissistic neuroticism to depression. Machiavellian agency is often overlooked in dark triad research. Further research should be undertaken to understand the mechanisms by which Machiavellian agency interacts with narcissistic extraversion to protect against depression.

## 1. Introduction

Personality is associated with various mental health outcomes (Aghababaei & Błachnio, 2015; Papageorgiou et al., 2019b) such as stress responses (Kajonius & Björkman, 2020; Ng et al., 2014), coping (Ng et al., 2014), and psychopathology severity (Aghababaei & Błachnio, 2015; Loeffler et al., 2020). Nevertheless, limited research exists investigating the influence of the dark triad on coping and psychopathology. The dark triad describes a group of three related but distinct personality traits (narcissism, Machiavellianism, and psychopathy; Paulhus and Williams (2002)) that share an antagonistic core. Even though distress is often experienced by those encountering high dark triad individuals (Y. Jiang et al., 2024), research suggests that high dark triad individuals also encounter feelings of emotional negativity in social interactions (Wei et al., 2025). Indeed, narcissistic individuals may undermine their wellbeing through aggression in response to ego threat, which not only harms others but also undermines their own wellbeing (Baumeister et al., 1996). Acquiring a nuanced understanding of which personality dimensions connect with psychopathology and coping methods will help to understand the antecedents and correlates of psychopathology.

### 1.1. The Dark Triad, Psychopathology, and Coping

Subclinical narcissism (referred to as ‘narcissism’ from this point on) reserves some of the characteristics of the personality disorder (narcissistic personality disorder), like grandiosity, entitlement, and superiority, but it is a stable disposition rather than a clinically relevant disorder. Subclinical narcissism has long been identified as a bi-dimensional trait (Wink, 1991), composed of grandiose and vulnerable narcissism, both with different nomological networks (Miller et al., 2021). Grandiose narcissism is characterized by confidence, other-oblivious willfulness, and exhibitionism (Egan et al., 2014). Researchers frequently report that grandiose narcissism is positively associated with wellbeing (Aghababaei & Błachnio, 2015; Jonason et al., 2013) and negatively associated with depression (Aghababaei & Błachnio, 2015; Jonason et al., 2013; Loeffler et al., 2020). However, vulnerable narcissism is characterized as more socially aversive and hyper-sensitive (Egan et al., 2014; Furnham, 2007). Vulnerable narcissism is associated positively with psychopathology (Papageorgiou et al., 2019b) and with more maladaptive coping patterns, like behavioral disengagement (Fernie et al., 2016), which is a tendency to experience an array of negative emotions such as depression, stress, and anxiety (Weiss & Miller, 2018). Recently, researchers have proposed more detailed compositions of narcissism, such as the three-factor model, which draws upon the five-factor model of personality (e.g., Collison et al., 2018). As such, the five-factor narcissism inventory (FFNI) was developed to measure the three factors (narcissistic extraversion, neuroticism, and antagonism). The FFNI measures the antagonistic core of trait narcissism and can help to explain what is unique to grandiose narcissism (linked to narcissistic extraversion) and vulnerable narcissism (linked to narcissistic neuroticism; Miller et al., 2016). Although one study demonstrated unidimensional associations between narcissism and coping and found that the trait is more often connected to task-focused coping (Birkás et al., 2016), there is a paucity of research connecting coping from a five-factor model perspective to coping methods.

Psychopathy is most often conceptualized as comprising two related but distinct dimensions, namely, primary and secondary psychopathy (Hosack et al., 2023; Loeffler et al., 2020). Individual differences in primary psychopathy are characterized by fearless dominance and stress immunity (Vidal et al., 2010). Secondary psychopathy is associated with neuroticism, poor self-regulation, and callousness (Vidal et al., 2010). M. Lyons et al. (2019) suggest that primary psychopathy, which is related to lower feelings of guilt (M. D. Lyons, 2016), could mitigate life stress through higher resilience. Findings such as these encourage further investigation into the dark triad dimensions with a focus on potential adaptivity. In terms of coping, one study found that psychopathy is associated with emotion-focused coping. However, this study did not look at psychopathy from a multidimensional perspective (Birkás et al., 2016).

Machiavellianism is characterized by strategic manipulation, cynicism, and a goal-oriented interpersonal style (Collison et al., 2018). However, its conceptualization and structure remain debated (Collison et al., 2018), with particular concerns about overlap between Machiavellianism and psychopathy (Truhan et al., 2021). To address these issues, Collison et al. (2018) developed the Five-Factor Machiavellianism Inventory (FFMI), which conceptualizes Machiavellianism as comprising three dimensions: agency (achievement-focused and self-confident), antagonism (callous and distrustful), and planfulness (deliberate and organized). This multidimensional approach aims to capture both the potentially adaptive and maladaptive aspects of Machiavellianism that were obscured in earlier unidimensional measures (Collison et al., 2018). Despite this advancement, few studies have examined how the FFMI dimensions relate to psychopathology. Earlier two-dimensional models suggest that the cynical, untrusting views component of Machiavellianism predicts multiple domains of psychopathology, even when controlling for manipulative interpersonal tactics (Monaghan et al., 2018). Finally, Machiavellianism was also, when measured unidimensionally, associated positively with emotion-focused coping (Birkás et al., 2016).

The five-factor model (FFM) research has consistently linked personality to psychopathology, including depression and anxiety (Collison et al., 2018; Ng et al., 2014). Therefore, understanding how specific FFM traits map onto the dark triad dimensions (e.g., Machiavellian agency) allows for a more nuanced theoretical account of which traits function adaptively or maladaptively in relation to psychopathology. Each dimension of the dark triad can be theorized in terms of its alignment with the Big Five traits—conscientiousness, extraversion, agreeableness, and neuroticism—with the exception of openness to experience, which has shown no significant associations with dark triad traits (Birkás et al., 2016; Collison et al., 2018). Drawing on the FFM, it can be hypothesized that Machiavellian agency, Machiavellian planfulness, narcissistic extraversion, and primary psychopathy may be adaptive in response to mental health symptoms, as these dimensions tend to associate positively with conscientiousness, extraversion, and/or agreeableness (Jourdy & Petot, 2017). Conversely, Machiavellian antagonism, narcissistic neuroticism, narcissistic antagonism, and secondary psychopathy are expected to be maladaptive, given their negative associations with conscientiousness, extraversion, and/or agreeableness or their positive associations with neuroticism (Jourdy & Petot, 2017).

Previous studies examining the dark triad and psychopathology support these hypotheses; for example, Machiavellian agency and planfulness correlate negatively with depression (Collison et al., 2018), indicating adaptive potential, whereas Machiavellian antagonism relates positively to depression, reflecting maladaptivity. Similarly, narcissistic extraversion has been found to relate negatively to depression (adaptive), while narcissistic antagonism and narcissistic neuroticism show positive associations (maladaptive; Miller et al., 2016). Finally, primary psychopathy is linked to emotional stability (adaptive), whereas secondary psychopathy correlates positively with anxiety (maladaptive; Vidal et al., 2010).

### 1.2. Network Analysis

By treating personality as an ecosystem of mutually affecting nodes, network analysis does not isolate and reduce complex systems down to latent variables or exclude the possibility of mutually influential associations and indirect associations (Costantini et al., 2015), which is pertinent when considering something as complex as psychopathology and coping. This focuses only on the data gathered, limiting assumptions, and can help to understand how the concepts coalesce. Network analysis has been used in previous dark triad and stress-related research and has yielded informative results. For example, Papageorgiou et al. (2019a) found that the dark triad can expound upon relationships in the prediction of stress above that which can be predicted by the Big Five. Specifically, given the many variables examined in the current study, it would be most effective to view it as one figure where variables can influence each other, and pathways can be observed. It is for these reasons that network analysis was chosen as the method of analysis in this study.

### 1.3. Aims and Hypotheses

We adopt a multidimensional approach to gain a comprehensive understanding of the adaptive and maladaptive aspects of the dark triad traits in relation to coping and psychopathology. Although existing research has evinced different associations among the dark triad traits, coping, and psychopathology (Aghababaei & Błachnio, 2015; Papageorgiou et al., 2019c), this will be the first study, to our knowledge, to do so with dark triad dimensions.

The aim of the current study is to, using network analysis, identify (mal)adaptive links among the dark triad dimensions and coping with psychopathology from an FFM perspective. This novel approach will give a comprehensive account of how the dark triad dimensions may help to protect an individual from psychopathology. By mapping the dark triad onto the FFM, we can consolidate which dimensions are likely to be adaptive and which are likely to be maladaptive. This will eventually direct research towards clinical samples and applications, like interventions to encourage behaviors associated with the adaptive personality dimensions.

Regarding psychopathology, we hypothesize that narcissistic extraversion will have negative connections to psychopathology (Hypothesis 1). Narcissistic antagonism and narcissistic neuroticism will have positive connections with psychopathology (Hypothesis 2). Primary and secondary psychopathy will exhibit negative and positive connections with psychopathology, respectively (Hypothesis 3). Last, Machiavellianism factors, including agency, planfulness, and antagonism, will have positive connections with psychopathology (Hypothesis 4). Coping methods will connect to the dark triad dimensions and to psychopathology (Hypothesis 5).

## 2. Method

### 2.1. Participants and Procedure

A priori power analysis was conducted to estimate the sample size necessary to test the study hypotheses (see the Supplementary Materials, Figure S1). The results indicated that a minimum of 350 participants would yield acceptable network accuracy, achieving over 0.7 in specificity, betweenness, and closeness and over 0.8 in correlation, strength, and sensitivity (Epskamp & Fried, 2018). Data were collected via an online recruitment platform, Prolific (www.prolific.co), with 371 participants initially participating. Participants were excluded if they did not complete the survey (*N* = 18), completed the survey in an unrealistic timeframe (i.e., <8 min; *N* = 3), or exhibited extreme acquiescence bias (*N* = 0). This resulted in a final sample size of 350 participants. Participants received GBP 3 as compensation for their time. There were 244 females (69.7%), 101 males (28.9%), and 5 non-binary (1.4%) participants. Most participants (139 people) were aged between 25 and 34 (39.7%), but this ranged from 18 to 65+. Participants were majority White (245), then Black (71). Participants read detailed information regarding the aims of the study and gave informed consent electronically. This study was approved by the University’s ethics committee (EPS 21_152). See the Supplementary Materials (Table S1) for further demographic information.

### 2.2. Measures

#### 2.2.1. Dark Triad Traits

Narcissism was measured using the Five-Factor Narcissism Inventory—Short Form (FFNI-SF; Sherman et al., 2015). The FFNI-SF uses 60 items on a 5-point Likert scale from ‘Disagree Strongly’ to ‘Agree Strongly.’ Narcissistic antagonism was calculated by summing the scores of the manipulativeness, exploitativeness, entitlement, lack of empathy, arrogance, reactive anger, distrust, and thrill-seeking facets. Narcissistic extraversion was measured by summing the scores for acclaim seeking, authoritativeness, grandiose fantasies, and exhibitionism. Finally, narcissistic neuroticism was measured by summing the scores of shame, indifference (reversed), and need for admiration. Reliability estimates for all scales are in Table 1.

Psychopathy was measured using the Levenson Self-Report Psychopathy (LSRP) Scale (Miller et al., 2008). The scale uses 26 questions on a Likert scale from ‘Disagree Strongly’ to ‘Agree Strongly.’ After reverse coding, the first 16 items were added together to constitute primary psychopathy, and the final 10 were added to constitute secondary psychopathy.

The Five-Factor Model Machiavellianism Inventory (FFMI; Collison et al., 2018) was utilized. This measure assesses three factors of Machiavellianism: agency, antagonism, and planfulness. It assesses 52 items on a Likert scale from 1 = ‘Disagree Strongly’ to 5 = ‘Agree Strongly.’ Within the agency factor, scales assessed achievement, activity, assertiveness, competence, invulnerability, and self-confidence. Antagonism assessed selfishness, immodesty, manipulativeness (low straightforwardness), callousness (low tendermindedness), and cynicism (low trust). Finally, planfulness assessed deliberation and order. Scores were calculated after reverse scoring by summing these facets.

#### 2.2.2. Coping with Stress

The Brief COPE Inventory (Collison et al., 2018) includes 28 items assessing 14 conceptually differing coping methods. Summing the two items for each coping facet provided the score for said coping facet. The facet scores were then added together in accordance with their coping domain: problem-focused coping (active coping, use of informational support, positive reframing, and planning), emotion-focused coping (emotional support, humor, acceptance, religion, and self-blame), and avoidance-focused coping (self-distraction, denial, substance use, and behavioral disengagement). The benefit of this scale is that it assesses adaptive and maladaptive coping methods.

The DASS (Depression, Anxiety, and Stress Scales) has been validated in a normative large non-clinical sample (Henry & Crawford, 2005). This study only examined depression, anxiety, and stress. Participants rated 21 items on a Likert scale from 0 = ‘Never’ to 3 = ‘Almost Always.’ Scoring was completed by summing all scores relevant to depression, anxiety, and stress, respectively, and then multiplying each by 2.

### 2.3. Statistical Analysis

As each question was a forced answer; there was no missing data. Initially, descriptive statistics were computed via RStudio (Posit Team, 2025; version 2025.5.0.496). To meet the assumption of multivariate normality, data was transformed to the multivariate normal using the ‘huge’ package (H. Jiang et al., 2020). Network estimation was conducted in RStudio (Posit Team, 2025) and the *bootnet* package (Epskamp et al., 2018) using the regularized EBICglasso estimation method. This approach minimizes spurious correlations, emphasizes unique pairwise interactions, and highlights potential mediations among the variables (Gojković et al., 2022; Epskamp et al., 2017). The network interpretation focused on centrality measures (strength, betweenness, and closeness) and node predictability. Network stability was detected via a case-dropping bootstrap, which iteratively re-estimated the network after randomly removing cases.

We obtained a correlation stability (CS) coefficient by correlating the original indices with the case-dropped indices. According to Epskamp et al. (2018), the CS coefficients for each centrality measure should preferably be over 0.5 but no lower than 0.25.

To discern the accuracy of the network analysis, non-parametric bootstraps were conducted (see Supplementary Materials, Figure S2). This technique creates a new dataset by resampling the data. The edge-weight bootstrap confidence intervals will then be compared to one another (Epskamp et al., 2018). If the confidence intervals were found to be large, this suggests that there is large differential variability across the network analysis, and it should be interpreted with caution. Finally, node predictability was calculated, representing the proportion of variance in a given node, explained by its connected nodes. Briganti et al. (2019) note that we assume that all edges for a node of interest are directed toward that node and that predictability estimates the influence all other nodes can have on the node of interest.

## 3. Results

Table 1 displays the descriptive statistics of the sample. The correlations between all variables can be seen in Table 2. There are notable, strong negative correlations between Machiavellian agency and depression, anxiety, and stress (r = −0.52, −0.22, and −0.36, respectively). On the other hand, narcissistic neuroticism and secondary psychopathy had strong positive correlations with psychopathology (e.g., r = 0.54 and 0.53 between depression and narcissistic neuroticism and secondary psychopathy, respectively).

### 3.1. Network Analysis of Dark Triad Factors, Psychopathology, and Coping

#### 3.1.1. Stability and Accuracy

Network stability tests examine the extent to which the indices can replicate across networks. We used the case-dropping bootstrap. Betweenness was 0.52, closeness was 0.67, and strength was 0.67. Therefore, the network analysis is relatively stable according to Epskamp et al. (2018).

We assessed the accuracy of the estimated network with 1000 cases. The appropriate graph can be examined in the Supplementary Materials, Figure S2. Some caution interpreting edges in the network analysis should be given, as there was just below half that extended across 0. If there are many that do not cut off at zero, this is a sign of instability (Epskamp et al., 2018). However, the edges for the main findings in this study did not all contain 0 (see Supplementary Details, Figure S2 for more details).

#### 3.1.2. Dark Triad Factors and Psychopathology

**Figure 1:**
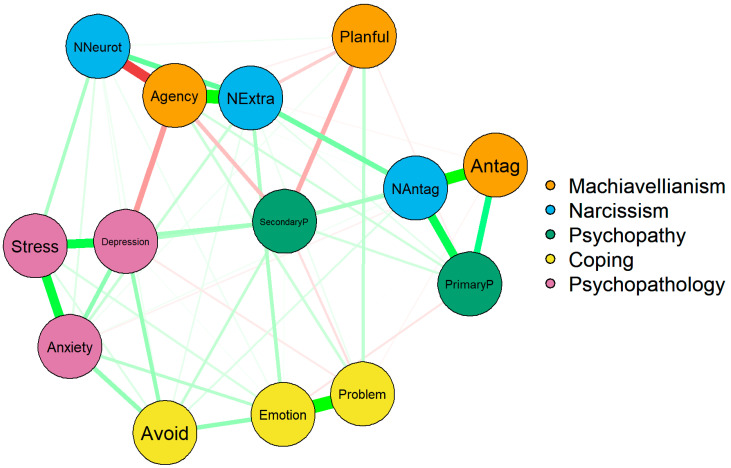
Network analysis of dark triad factors, psychopathology, and coping. Note: network analysis of the structure of associations between dark triad factors with psychopathology and coping methods. NAntag—narcissistic antagonism (FFNI); NExtra—narcissistic extraversion (FFNI); NNeurot—narcissistic neuroticism; PP—primary psychopathy (LSRP); SP—secondary psychopathy (LSRP); Problem—problem-focused coping (BriefCOPE); Emotion—emotion-focused coping (BriefCOPE); Avoid—avoidance coping (BriefCOPE); Antag—antagonism (FFMI); Planful—planfulness (FFMI). Green represents positive edges, and red represents negative edges.

As seen in Figure 1, Machiavellian agency and depression had a strong negative connection. Machiavellian agency was the only personality variable to negatively connect to a psychopathology measure. Narcissistic neuroticism was connected positively to all psychopathology variables, although only weakly connected with depression. Narcissistic extraversion was positively connected to anxiety but was not connected with any other psychopathology measure. Machiavellian antagonism showed a weak negative connection with anxiety. Secondary psychopathy was positively connected to depression.

#### 3.1.3. Dark Triad Factors, Coping, and Psychopathology

Avoidance-focused coping, a more maladaptive coping method, connected positively to all psychopathology measures. Emotion and problem-focused coping, which are by and large seen as a more adaptive coping methods, were connected positively to narcissistic extraversion and Machiavellian agency, respectively. However, there was a positive connection between emotion-focused coping and anxiety.

#### 3.1.4. Dark Triad Factors—Centrality

**Figure 2:**
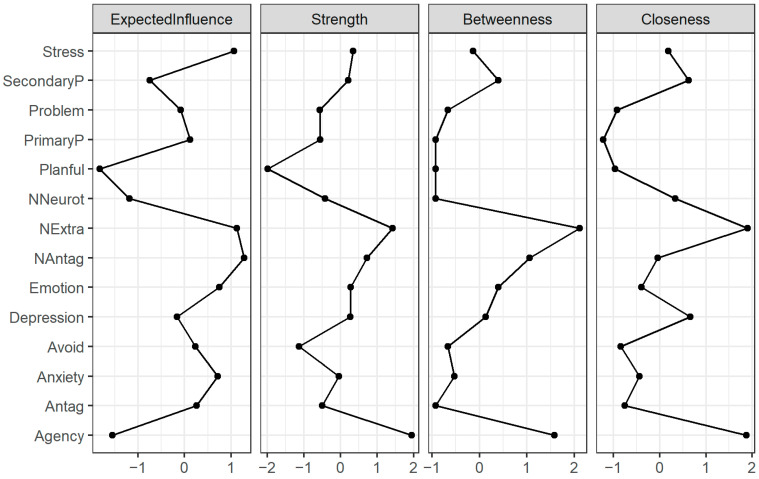
Centrality indices of network 1. Note: centrality indices of all variables. Agency and grandiose narcissism showed the highest centrality, and planfulness showed the lowest. NAntag—narcissistic antagonism (FFNI); NExtra—narcissistic extraversion (FFNI); NNeurot—narcissistic neuroticism; PP—primary psychopathy (LSRP); SP—secondary psychopathy (LSRP); Problem—problem-focused coping (BriefCOPE); Emotion—emotion-focused coping (BriefCOPE); Avoid—avoidance coping (BriefCOPE); Antag—antagonism (FFMI); Planful—planfulness (FFMI).

The most central node was agency and narcissistic extraversion, showing the highest strength, betweenness, and closeness. This indicates that these nodes not only quickly influence other nodes but are also quickly influenced by other nodes in the network (Figure 2). This means that Machiavellian agency and narcissistic extraversion frequently lie on the shortest paths between other nodes.

#### 3.1.5. Predictability

Stress was best predicted by the network (Figure 1) at 0.67, as seen in Table 3. This means that nodes connected to stress predict 67% of the variance in the stress node. Depression can also be predicted from this network (0.65). Interestingly, avoidance-focused coping was least predictable in the network (0.41). This suggests that the data is better at predicting adaptive coping methods compared to maladaptive coping methods.

#### 3.1.6. Expected Influence

Predictability does not consider the direction of the edges, so to gain more insight, we calculated the expected influence of each node, as seen in Figure 2. Agency showed the least expected influence. Given the previous centrality findings, this means that it is not influencing the nodes but rather being influenced by other nodes. Narcissistic antagonism presented the highest expected influence, indicating that it has an influential position in the network (Figure 2). Avoidance and emotion-focused coping also showed high expected influence, highlighting that coping methods are important in understanding the connections between personality and psychopathology.

## 4. Discussion

The current study sought to address the gap in understanding the connections between the dark triad and psychopathology from an FFM perspective. Through self-report and network analysis, we examined the associations between the dark triad dimensions from an FFM perspective with coping and psychopathology. We predicted that narcissistic extraversion would have a negative connection to psychopathology (Hypothesis 1), while narcissistic antagonism and narcissistic neuroticism would have positive connections to psychopathology (Hypothesis 2). Hypothesis 1 was fully supported, and Hypothesis 2 was partially supported. There were no direct connections between narcissistic antagonism and psychopathology. In terms of coping, narcissistic extraversion was the most adaptive, as it was the only narcissism factor to connect positively with emotion-focused coping.

We predicted that primary and secondary psychopathy would exhibit negative and positive connections with psychopathology, respectively (Hypothesis 3). This hypothesis is not supported in the present study, as there were no connections between psychopathy and psychopathology. Secondary psychopathy was more maladaptive in terms of coping compared to primary psychopathy, as it was weakly and negatively connected to both emotion- and problem-focused coping. This suggests that someone scoring highly in secondary psychopathy may be less likely to use these coping methods when faced with a stressor.

Last, we predicted that Machiavellianism factors, including agency, planfulness, and antagonism, would have positive connections with psychopathology, which was not supported by the data (Hypothesis 4). However, the current study identified a novel connection regarding Machiavellian agency. Machiavellian agency operates a strategic position in the network analysis (high centrality), but low expected influence implies that it operates as a bridge rather than an influencer. More specifically, we observed a positive connection from narcissistic extraversion and a negative connection from narcissistic neuroticism to Machiavellian agency, respectively, and a negative connection from agency to depression. Taken together, this suggests that there may be an adaptive pathway where narcissistic extraversion acts on depression through Machiavellian agency. In behavioral terms, individuals high in narcissistic extraversion tend to seek social approval and assertiveness (Sherman et al., 2015). When this orientation is combined with Machiavellian agency, they may strategically manage social situations, regulate self-presentation, and maintain a sense of effectiveness and control. These strategic behaviors could confer a protective function against negative affect, thereby reducing vulnerability to depressive symptoms.

### 4.1. Coping

A recent development in network analysis is the use of predictive path models (Borsboom et al., 2021; Golino & Epskamp, 2017). This has the potential to identify different nodes working together to act on an outcome node (Borsboom et al., 2021). For example, narcissistic extraversion and neuroticism acted positively and negatively, respectively, on depression through Machiavellian agency. Furthermore, all four nodes are high in centrality, suggesting that they are likely to influence or be influenced by the other nodes in the network. Given that agency did not exert influence on depression, we propose that it acts as a crucial bridge between narcissistic neuroticism and extraversion to depression. This is congruent with the previous literature, whereby grandiose narcissism is connected negatively to depression (Aghababaei & Błachnio, 2015; Jonason et al., 2013; Loeffler et al., 2020). The design of this study offers nuances in understanding many complex variables without assuming latent variables. The finding that Machiavellian agency is influential in this connection is, to our knowledge, novel.

Examining the centrality measures, specifically predictability and expected influence, revealed further support for the claim that narcissistic extraversion (positively) and neuroticism (negatively) are acting on depression through agency (negatively). Depression was highly predictable, suggesting that its connected nodes, agency, psychopathology, secondary psychopathy, and avoidance-focused coping can predict variance in depression well. However, predictability does not consider the direction of the edge (Costantini et al., 2015). Therefore, we examined expected influence, which does consider the direction of the edge. When we did, agency was one of the lowest nodes in terms of expected influence. This suggests that it is not acting on the other nodes but is instead being influenced by the other nodes. This, along with the lack of direct connection between narcissistic extraversion and depression, highlights that agency operates a strategic position in a network of the dark triad and psychopathology. This is, to the author’s knowledge, a novel finding and highlights that Machiavellian agency could be a bridge between personality and coping and psychopathology, emphasizing the psychological function of Machiavellian agency in everyday behavior.

### 4.2. Limitations and Future Directions

This study shares limitations with many other studies in the area; nonetheless, they must be considered. First, the use of self-report measures introduces potential recall, social desirability, and common method bias. Additionally, the data were skewed towards white female participants, potentially masking gender and country-related nuances (Jonason et al., 2020). Future research should extend these findings by explicitly examining sex and country-based differences.

A further consideration concerns the conceptual overlap between narcissistic extraversion and Machiavellian agency, which were highly correlated. At the surface level, there are similarities in the acclaim facet (from narcissistic extraversion) and the achievement facet (from Machiavellian agency), which could help to explain the strong connection. Indeed, Grabovac and Dinić (2022) note that the FFM profile of the FFMI was more similar to expert Machiavellianism profiles than expert narcissism. Where Machiavellian agency differs from narcissistic extraversion is that Machiavellian agency contains an invulnerability facet that focuses on emotional stability. This expounds why Machiavellian agency drives the connection between personality and psychopathology. Future research should still take this into consideration when using the FFM scales.

Coping research also carries contextual limitations. Self-reported coping strategies may not be uniformly adaptive across situations; for instance, problem-focused coping may be less effective in response to grief. Coping flexibility—the capacity to discontinue ineffective strategies and adopt more appropriate ones (Kato, 2015)—may, therefore, represent a more accurate indicator of psychological adjustment. If we were to understand how a person can cope flexibly within their specific situation, it would help clinicians understand how to best treat their patients, given their natural predispositions to certain coping methods and their personality. To this end, subsequent studies should strive to understand how coping flexibility relates to the dark triad.

While network analysis can identify patterns of co-occurrence and potential pathways of influence, it cannot determine whether one variable causes a change in another. However, this limitation should be balanced against the strengths of network analysis, specifically, its minimal reliance on latent constructs and a priori assumptions about the structure of relationships. Given the limited existing studies addressing how the dark triad dimensions, conceptualized through the five-factor model, relate to mental health and coping, it was methodologically appropriate to first map these associations broadly. This data-driven approach provides a necessary foundation for future confirmatory work using longitudinal or experimental designs, such as structural equation modeling, to test causal hypotheses.

### 4.3. Implications for Theory and Practice

In this study, we highlight which personality factors can be adaptive in association with psychopathology. This could be applied to future research, whereby the utilization of idiographic networks can identify whether a person has an increased likelihood of experiencing psychopathology in part due to their personality and preferred coping methods. Clinicians and psychologists could use this to tailor psychotherapy, provide contemporaneous feedback to patients, and make long-term predictions. This takes on greater significance when we understand how many commonly used treatments (like Cognitive Behavioral Therapy) are not equally effective for all clients (Mansueto et al., 2022). Therefore, having individually tailored software that considers personality and context could allow clinicians to determine the treatment program that is likely to be best suited to that individual.

## 5. Conclusions

These findings expand our understanding of how psychopathology and coping methods relate to the dark triad traits, particularly highlighting the adaptive and maladaptive aspects of lower-order dark triad factors. To the author’s knowledge, for the first time, Machiavellianism was assessed at the factor level with psychopathology and coping with stress using network analysis. This indicated that Machiavellian agency could play a key role in protecting an individual from psychopathology by acting as a bridge where more narcissistic extraversion and less narcissistic neuroticism negatively affect depression through agency. Future research should study coping dynamically and not rely on static coping methods.

## Figures and Tables

**Table 1:** Descriptive statistics.

Factor	Mean (SD)	Variance	Skew	Kurtosis	α
Narcissism					
Antagonism	72.64 (19.19)	369.23	0.54	0.21	0.91
Extraversion	47.50 (12.69)	161.05	−0.09	−0.65	0.89
Neuroticism	38.16 (8.51)	72.42	−0.24	−0.49	0.81
Machiavellianism					
Agency	76.03 (16.65)	277.17	−0.12	−0.41	0.90
Antagonism	47.78 (11.64)	135.55	0.75	0.81	0.87
Planfulness	29.14 (4.67)	21.75	−0.41	0.25	0.76
Psychopathy					
Primary	33.28 (7.96)	63.41	0.62	−0.03	0.88
Secondary	22.27 (4.91)	24.15	0.32	−0.16	0.74
Coping					
Problem	20.76 (5.37)	28.89	−0.16	−0.38	0.85
Avoidance	14.60 (4.18)	17.47	0.75	0.06	0.73
Emotion	26.81 (5.87)	34.42	0.02	−0.31	0.71
Psychopathology					
Depression	27.67 (10.50)	110.15	0.67	−0.3	0.92
Anxiety	23.81 (8.51)	72.46	0.81	0.05	0.85
Stress	28.76 (8.58)	73.54	0.30	−0.2	0.85

**Table 2:** Spearman’s intercorrelation matrix for all variables.

	1	2	3	4	5	6	7	8	9	10	11	12	13
1. M agency													
2. M antag	0.10												
3. M plan	−0.11 *	−0.20 **											
4. Primary P	0.24 **	0.71 **	−0.28 **										
5. Secondary P	−0.32 **	0.47 **	−0.30 **	0.42 **									
6. N antag	0.22 **	0.78 **	−0.24 **	0.75 **	0.48 **								
7. N extra	0.63 **	0.30 **	−0.24 **	0.43 **	0.09	0.50 **							
8. N neurot	−0.59 **	0.08	0.03	−0.02	0.35 **	0.08	−0.06						
9. Problem	0.28 **	−0.08	0.15 **	−0.07	−0.17 **	0.05	0.29 **	−0.07					
10. Emotion	0.09	0.03	−0.02	−0.00	0.14 **	0.18 **	0.33 **	0.17 **	0.60 **				
11. Avoid	−0.19 **	0.22 **	−0.13 *	0.22 **	0.40 **	0.30 **	0.21 **	0.34 **	0.06	0.37 **			
12. Depression	−0.52 **	0.17 **	−0.04	0.09	0.53 **	0.17 **	−0.10	0.54 **	−0.11 *	0.24 **	0.55 **		
13. Anxiety	−0.22 **	0.16 **	−0.04	0.16 **	0.40 **	0.28 **	0.20 **	0.46 **	0.12 *	0.40 **	0.58 **	0.66 **	
14. Stress	−0.36 **	0.18 **	−0.07	0.15 **	0.53 **	0.26 **	0.07	0.53 **	0.05	0.33 **	0.55 **	0.76 **	0.76 **

Note: * = *p* < 0.05; ** = *p* < 0.01. M = Machiavellianism; antag = antagonism; plan = planfullness; P = psychopathy; N = narcissism; extra = extraversion; neurot = neuroticism; Problem = problem-focused coping; Emotion = emotion-focused coping; Avoid = avoidance-focused coping.

**Table 3:** Variables and their associated predictability.

Variable	Predictability
Stress	0.67
Depression	0.65
Anxiety	0.59
Emotion-focused coping	0.50
Problem-focused coping	0.42
Avoidance-focused coping	0.41

Note: predictability of network 1. Predictability values could range from 0 to 1, where higher values indicate better prediction of the node by the data.

## Data Availability

Data are available upon request.

## Supplementary Materials

The following supporting information can be downloaded at https://www.mdpi.com/article/10.3390/bs15121617/s1, Figure S1: A power analysis was conducted to determine the required sample size to test our hypotheses. The results suggested that 350 participants would be sufficient to achieve over 0.6 in specificity and, betweenness and over 0.8 in closeness, correlation, strength, and sensitivity, which is acceptable (Epskamp & Fried, 2018); Table S1: Demographic information of the sample.; Figure S2: Non-parametric bootstrap figure to examine the ac-curacy of the data. To discern the accuracy of the network analysis, non-parametric bootstraps were conducted (see Supplementary Materials). This technique creates a new dataset by resampling the data. The edge-weight bootstrap confidence intervals will then be compared to one another (Epskamp et al., 2018). If the confidence intervals were found to be large, this suggests that there is large differential variability across the network analysis, and it should be interpreted with caution.

## Abbreviations

The following abbreviations are used in this manuscript:

FFNI	Five-Factor Narcissism Inventory
FFMI	Five-Factor Machiavellianism Inventory
FFNI-SF	Five-Factor Narcissism Inventory – Short Form
M	Machiavellianism
Antag	Antagonism
Plan	Planfullness
P	Psychopathy
N	Narcissism
Extra	Extraversion
Neurot	Neuroticism
Problem	Problem-focused coping
Emotion	Emotion-focused coping
Avoid	Avoidance-focused coping
