# Supplementary material for: More than Just Aversive: A Network Analysis of the Dark Triad, Coping, and Psychopathology
Source: Behav Sci (Basel). 2025 Nov 24;15(12):1617. doi: 10.3390/bs15121617 (PMC12729700; doi:10.3390/bs15121617)
Supplement: Supplementary file 1 [file behavsci-15-01617-s001.zip › behavsci-3916539-supplementary.pdf]

## Supplementary Materials

**Figure S1.** Evidence of Power Analysis.

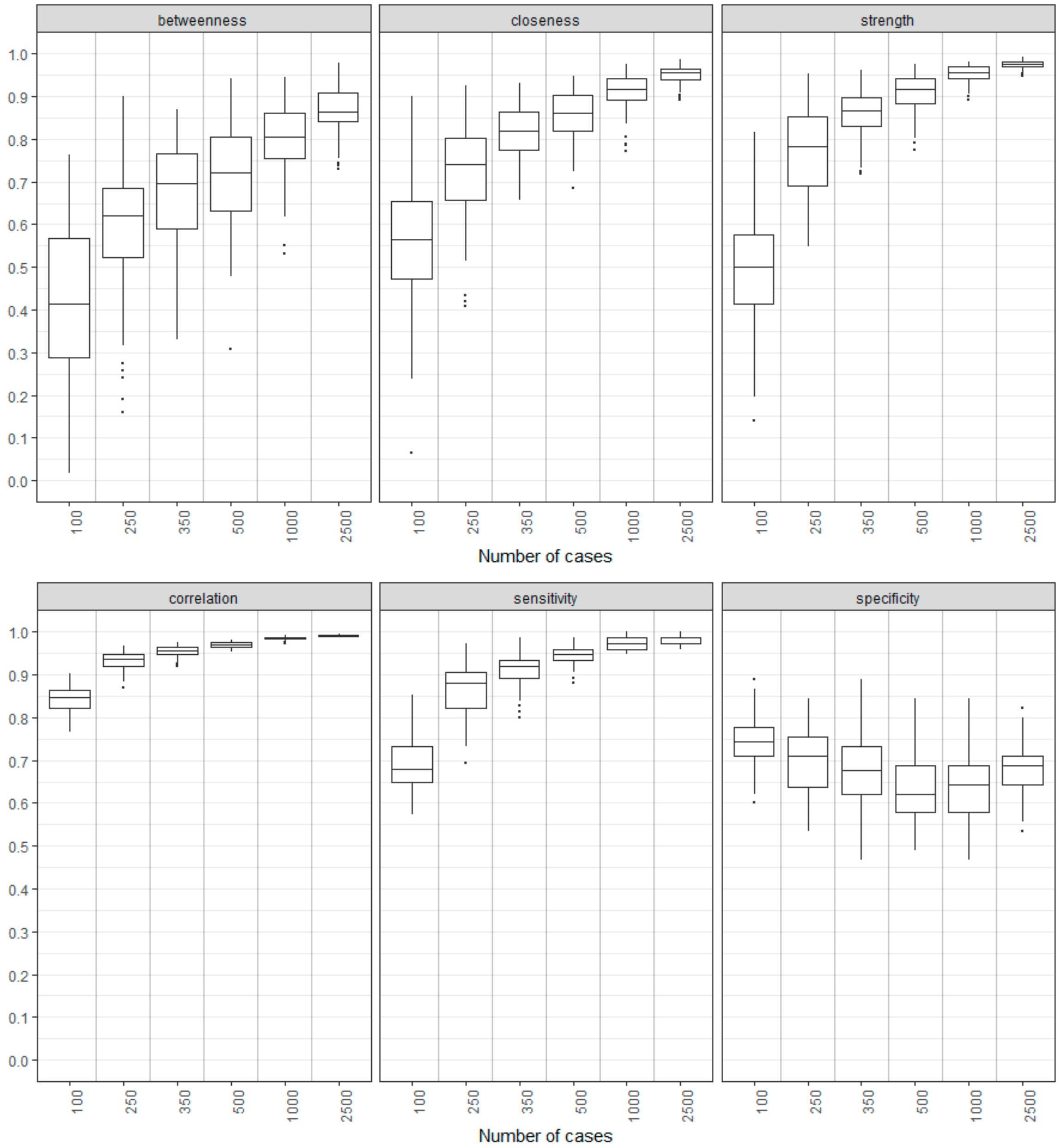


**Figure S1.** A power analysis was conducted to determine the required sample size to test our hypotheses. The results suggested that 350 participants would be sufficient to achieve over 0.6 in specificity and betweenness and over 0.8 in closeness, correlation, strength, and sensitivity, which is acceptable (Epskamp & Fried, 2018).

**Table S1.** Demographic information of the sample.

| Ethnicity | Count (%) |
|---|---|
| White | 245 (70) |
| Black | 71 (20.2) |
| Mixed | 14 (4) |
| Other | 10 (2.86) |
| Asian | 8 (2.29) |
| Prefer not to say | 2 (0.57) |
| Employment | |
| Full time | 146 (41.7) |
| Prefer not to say | 66 (18.86) |
| Part time | 53 (15.14) |
| Not in paid work (e.g., homemaker, retired, or disabled) | 37 (10.57) |
| Unemployed (and job seeking) | 30 (8.57) |
| Other | 15 (4.29) |
| Due to start a new job within the next month | 3 (0.86) |
| Gender | |
| Female | 244 |
| Male | 101 |
| Non-binary/third gender | 5 |

**Figure S2.** Accuracy of the Network Edges

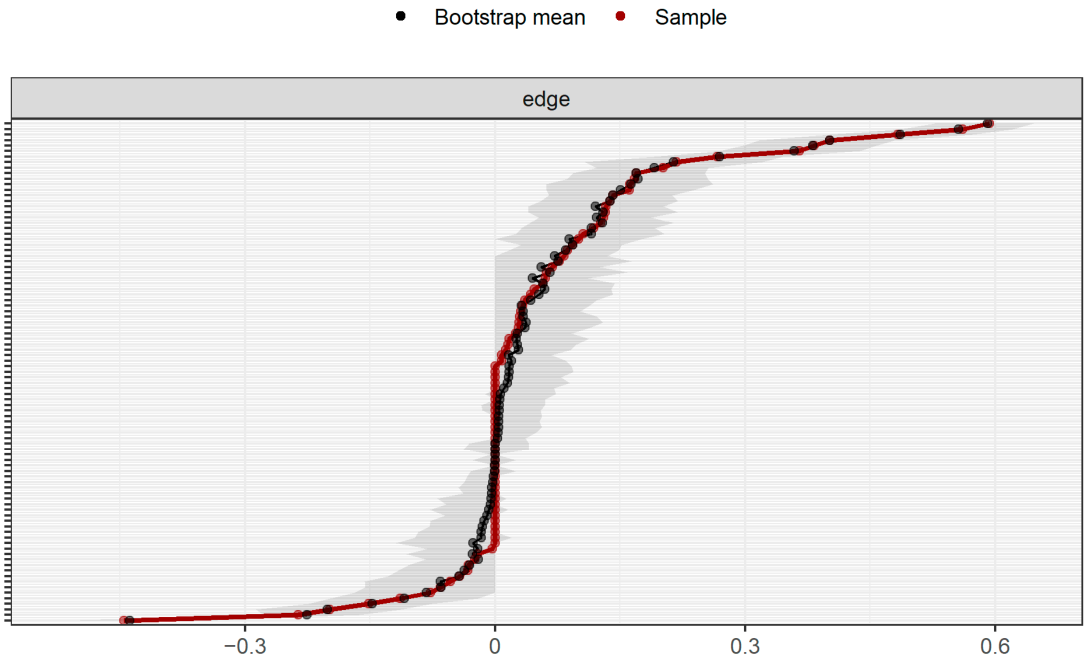


.

**Figure S2.** Non-parametric bootstrap figure to examine the accuracy of the data. To discern the accuracy of the network analysis, non-parametric bootstraps were conducted. This technique creates a new dataset by resampling the data. The edge-weight bootstrap confidence intervals will then be compared to one another (Epskamp et al., 2018). If the confidence intervals were found to be large, this suggests that there is large differential variability across the network analysis, and it should be interpreted with caution.
